# Combination of baseline metabolic tumour volume and early response on PET/CT improves progression-free survival prediction in DLBCL

**DOI:** 10.1007/s00259-016-3315-7

**Published:** 2016-02-23

**Authors:** N. George Mikhaeel, Daniel Smith, Joel T. Dunn, Michael Phillips, Henrik Møller, Paul A. Fields, David Wrench, Sally F. Barrington

**Affiliations:** Department of Clinical Oncology, Guy’s and St Thomas’ NHS Foundation Trust, Westminster Bridge Road, SE1 7EH London, UK; PET Imaging Centre at St Thomas’ Hospital, Division of Imaging Sciences and Biomedical Engineering, King’s College London, London, UK; Department of Cancer Epidemiology and Population Health, King’s College London, London, UK; Department of Haematology, Guy’s and St Thomas’ NHS Foundation Trust, London, UK

**Keywords:** Positron emission tomography, Diffuse large B-cell lymphoma, Metabolic tumour volume, Deauville score, Response assessment

## Abstract

**Background:**

The study objectives were to assess the prognostic value of quantitative PET and to test whether combining baseline metabolic tumour burden with early PET response could improve predictive power in DLBCL.

**Methods:**

A total of 147 patients with DLBCL underwent FDG-PET/CT scans before and after two cycles of RCHOP. Quantitative parameters including metabolic tumour volume (MTV) and total lesion glycolysis (TLG) were measured, as well as the percentage change in these parameters. Cox regression analysis was used to test the relationship between progression-free survival (PFS) and the study variables. Receiver operator characteristics (ROC) analysis determined the optimal cut-off for quantitative variables, and Kaplan–Meier survival analysis was performed.

**Results:**

The median follow-up was 3.8 years. As MTV and TLG measures correlated strongly, only MTV measures were used for multivariate analysis (MVA). Baseline MTV (MTV-0) was the only statistically significant predictor of PFS on MVA. The optimal cut-off for MTV-0 was 396 cm^3^. A model combing MTV-0 and Deauville score (DS) separated the population into three distinct prognostic groups: good (MTV-0 < 400; 5-year PFS > 90 %), intermediate (MTV-0 ≥ 400+ DS1-3; 5-year PFS 58.5 %) and poor (MTV-0 ≥ 400+ DS4-5; 5-year PFS 29.7 %)

**Conclusions:**

MTV-0 is an important prognostic factor in DLBCL. Combining MTV-0 and early PET/CT response improves the predictive power of interim PET and defines a poor-prognosis group in whom most of the events occur.

## Introduction

The cure rate of diffuse large B-cell lymphoma (DLBCL) has improved over the last two decades with the addition of rituximab to CHOP chemotherapy (RCHOP) and improvements in dose intensity and supportive care [1, 2]. Nonetheless, a proportion of patients are not cured with RCHOP, either due to primary refractory disease or later relapse following an initial response. Salvage treatments after RCHOP are less effective than after CHOP, probably because the increased cure rate with upfront treatment leaves a worse prognostic group for salvage [3, 4]. Early identification of patients unlikely to be cured with RCHOP is an important step towards testing alternative approaches in order to improve their chance of cure.

18 F-fluorodeoxyglucose (FDG) PET/CT performed early during a course of chemotherapy (interim PET, iPET) shows early response and has been found to be prognostic in Hodgkin lymphoma [5–9]. This has led to several clinical studies testing response-adapted treatment algorithms. However in DLBCL, iPET studies have shown conflicting results [10–21]. Even in studies showing a significant difference between early complete metabolic response (CMR) and partial metabolic response (PMR), the progression-free survival (PFS) in the partial response group has remained too high, around 50 % at 2–5 years, to consider early change of treatment. More recent response criteria, e.g. Deauville score (DS) and the reduction in the maximum SUV (ΔSUVmax), have not increased the positive predictive value of iPET in DLBCL, and there is a need to improve the current response criteria by exploring additional parameters to improve the predictive ability of interim PET.

In addition to early treatment response, baseline characteristics of the lymphoma, including tumour burden, impact significantly on outcome [22]. Quantitative imaging methods using PET/CT to assess total tumour burden are under development and may be more predictive than existing methods [23]. We hypothesised that patients may achieve a good early metabolic response but their chance of cure may be different based on pre-treatment tumour burden. The objectives of this study were to assess the prognostic value of quantitative PET measurements, particularly metabolic tumour burden, and to test whether combining measurements of metabolic tumour burden at baseline with early PET response could improve the prognostic ability of iPET in DLBCL.

## Patients and methods

### Study population

This is a retrospective study including 147 consecutive patients treated at Guy’s and St Thomas’ Hospital, London, between March 2005 and August 2012, according to a prospectively agreed-upon departmental protocol. The study was approved by the hospital review committee, and all patients gave written informed consent. The inclusion criteria were as follows: histologically proven DLBCL, treatment with RCHOP chemotherapy, PET/CT scans before and after two cycles of chemotherapy, and a minimum follow-up of 12 months. Exclusion criteria were coexistent low-grade lymphoma, other malignancy or active sarcoidosis, previous anthracycline chemotherapy, and no assessable disease on baseline PET/CT.

### Treatment protocol

**Fig. 1:**
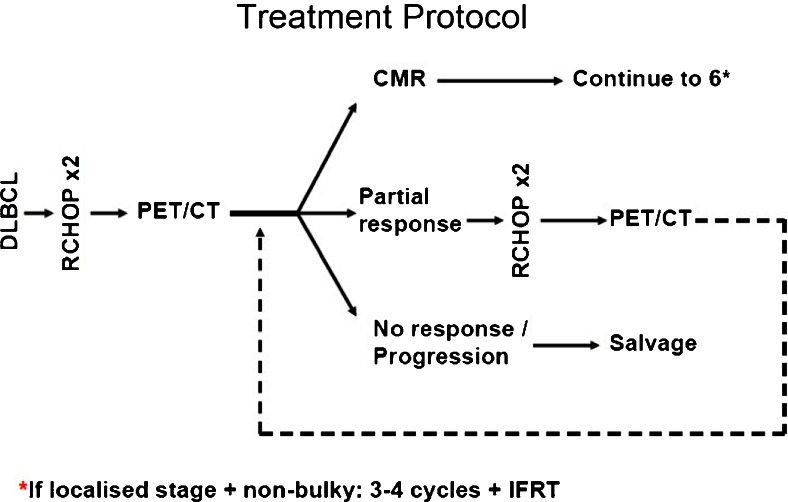
Treatment protocol. CMR = complete metabolic response (defined as Deauville score 1–3). Partial response was defined as Deauville score 4–5 with improvement compared to baseline. No response/progression was defined as Deauville score 4–5 with no improvement or progression compared to baseline

All patients received standard-dose RCHOP chemotherapy [1]. Figure 1 summarises the treatment algorithm. PET/CT scan after two cycles (iPET2) was not used to change treatment unless there was definite evidence of progression or lack of response, taking into consideration all clinical and laboratory information. Patients with CMR continued to a total of 6 cycles of chemotherapy or 3–4 cycles followed by involved-field radiotherapy in the case of stage I or II non-bulky disease. Patients with PMR after two cycles received two further cycles of RCHOP followed by repeat PET/CT scan (iPET4). If iPET4 showed CMR or further response with residual uptake (PMR), treatment continued to six cycles. All patients had an end-of-treatment scan (CT if there was CMR on iPET; PET/CT if there was PMR on iPET). Consolidation radiotherapy was administered according to departmental protocol and clinician discretion.

In total, seven patients changed treatment due to progressive disease while on RCHOP. Two patients progressed after cycle 2: one patient with rising lactate dehydrogenase (LDH) and no significant response on PET, and a second patient showing progression of disease on PET. Both were changed to salvage chemotherapy but progressed and died. Two other patients progressed after three cycles: one with recurrence of palpable bulky lymphadenopathy and another with cytologically proven CNS relapse. Both were changed to salvage chemotherapy but progressed and died. Two patients progressed after cycle 4, both with increased FDG uptake: one was successfully salvaged with further chemotherapy and radiotherapy, and one died of progressive disease following salvage high-dose chemotherapy and stem cell transplant. Finally, one patient progressed after cycle 5 with increasing disease bulk on CT, which was biopsy-proven, and died following salvage chemotherapy.

With regard to radiotherapy (RT), 50 patients had RT. In 35 patients, RT was planned treatment as part of a combined treatment approach for stage I or II disease (relapse = 1/35). Of the 15 with stage III or IV disease who received RT, three had stage III disease and received RT to a residual focus of uptake (relapse = 0/3), and 12 had stage IV disease and received RT for the following reasons: site of bony disease (two), contralateral testicular RT (one), residual activity on PET-6 (four), initial bulk and DS 3 on PET-6 (two), initial bulk and residual mass (one), clinician discretion (two) (relapse = 1/12).

### PET/CT scanning

PET/CT scans were performed after a 6-h fast. Images were acquired from skull base to upper thighs 90 minutes after administration of 370 MBq of 18 F-FDG using DST or VCT scanners (GE Healthcare, Waukesha, WI, USA). Baseline and interim scans were performed on the same scanner. Quality control procedures to ensure accurate quantitation developed for clinical trials have been previously described [24]. Paired scans were displayed using a fixed SUV scale and colour table, and were reviewed by an experienced nuclear medicine physician (SFB), who was blinded to clinical outcome and other baseline parameters such as the International Prognostic Index (IPI). The Deauville score was allotted by comparing the uptake in the residual lesion with the highest uptake, if present, with the intensity of uptake in normal mediastinum and liver. A score of 5 was given if the maximum SUV in the tumour was ≥3 times greater than the maximum SUV in a large region of normal liver.

In-house software developed by MP was used to automatically segment tumour volumes with SUV ≥2.5. Volumes were edited by SFB to remove physiological uptake, e.g. myocardium adjacent to mediastinal nodal disease. All tumour volumes were summed to calculate the total metabolic tumour volume (MTV). Tumour lesion glycolysis (TLG) was calculated as MTV × mean SUV in the volume.

The following parameters were determined on the baseline scan: SUVmax-0, MTV-0 and TLG-0. The following parameters were determined on the scan after two cycles: SUVmax-2, MTV-2, TLG-2 and the Deauville score. Changes in these parameters during treatment were also calculated: % change in SUVmax (ΔSUVmax), % change in MTV (ΔMTV), % change in TLG (ΔTLG).

### Statistical analysis

The endpoint of the study was PFS, defined as the time from diagnosis to the point of progression or death from any cause. Patients still alive were censored at the date of last contact.

To determine which measures were predictive of PFS, univariate Cox regression (CR) analysis was performed on all quantitative parameters as well as IPI and Deauville score. The IPI score was analysed according to the standard prognostic groups: low risk (0–1), low intermediate (2), high intermediate (3), and high (4–5). Continuous measures were grouped into tertiles. Variables which were significantly associated with PFS in univariate analysis were included in multivariate analysis in order to identify measures independently predictive of PFS.

Receiver operator characteristics (ROC) analysis was performed on continuous variables to determine optimal cut-off values. The effect of individual parameters on PFS was studied with Kaplan–Meier (KM) survival analysis using dichotomous grouping based on ROC analysis. IPI scores were grouped into 0–1 and 2–5, and DS scores into 1–3 (CMR) and 4–5 (incomplete response). For ΔSUVmax, we used the previously reported cut-off of 66 % [22, 28].

Based on these results, a prognostic model combining baseline and response parameters was constructed on which KM survival analysis was performed.

Statistical significance was considered at *p* < 0.05. Data were analysed using SPSS software (IBM Corp. Released 2011. IBM SPSS Statistics for Windows, Version 20.0. Armonk, NY: IBM Corp.).

## Results

The median follow-up for 147 patients was 3.8 years (range 1.3–7.9 years). The clinical characteristics are summarised in Table 1. The 5-year PFS and OS rates for the whole group were 65.4 % and 73.7 %, respectively. Table 2 shows the descriptive statistics for the quantitative PET parameters.

**Table 1:** Patient characteristics

Patient characteristics		*n* = 147 (%)
Sex	Female	74
	Male	73
Age	Range	22–86
	Median	57
	≥60 years	71 (48 %)
Performance status		
	0–1	103 (70 %)
	2–4	44 (30 %)
Raised LDH		93 (63 %)
Extra-nodal sites ≥ 2		73 (50 %)
Bulky disease (≥10 cm)		59 (40 %)
Stage	I	17 (11 %)
	II	29 (20 %)
	III	16 (11 %)
	IV	85 (58 %)
IPI	0/1	45 (31 %)
	2	18 (12 %)
	3	38 (26 %)
	4/5	46 (31 %)

IPI = International Prognostic Index, LDH = lactate dehydrogenase

**Table 2:** Mean, median and range of quantitative PET parameters

	Mean	Median	Min	Max
MTV-0 (cm^3^)	990.14	595.12	1.50	7357.20
TLG-0 (cm^3^)	6815.91	4669.52	5.69	36,570.00
SUVmax-0	27.89	27.25	5.38	110.52
MTV-2 (cm^3^)	34.97	0.59	0.00	1608.70
TLG-2 (cm^3^)	213.45	1.64	0.00	13,135.00
SUVmax-2	2.05	2.73	0.00	10.34
ΔMTV (%)	−95.81	−99.95	−100.00	−6.95
ΔTLG (%)	−96.88	−99.98	−100.00	−31.71
ΔSUVmax (%)	−69.03	−70.21	−100.00	41.39

Univariate CR analysis (Table 3) showed that the following variables were statistically significant: IPI, MTV-0, TLG-0, DS, MTV-2, TLG-2, SUVmax-2 and ΔSUVmax.

**Table 3:** Cox regression analysis

	LEVELS	CASES (*n* = 147)	UNIVARIATE				MULTIVARIATE			
			HR		95 % CI for HR		HR		95 % CI for HR	
IPI groups	(0,1)	45		1.00				1.00		
	2	18		4.23	1.34	13.34		2.95	0.82	10.60
	3	38		4.74	1.73	12.98		2.30	0.74	7.21
	(4,5)	46		5.49	2.07	14.53		2.98	0.98	9.08
		*TREND:*	***χ2 =***	***12.39***	***P-value =***	***.0004***	***χ2 =***	***2.78***	***P-value =***	***.0955***
**DS**	1	34		1.00				1.00		
	2	18		0.74	.234	2.321		0.21	0.04	1.09
	3	30		0.49	.169	1.402		0.06	0.01	0.42
	4	47		1.33	.621	2.844		0.09	0.01	0.62
	5	18		3.86	1.725	8.640		0.23	0.03	1.88
		***TREND:***	***χ2 =***	***7.61***	***P-value =***	***.0058***	***χ2 =***	***0.95***	***P-value =***	***.3303***
**SUVmax-0**	Lower	49		1.00						
Tertiles	Middle	49		1.44	.74	2.79				
	Upper	49		0.86	.41	1.78				
		***TREND:***	***χ2 =***	***0.17***	***P-value =***	***.6831***				
**MTV-0**	Lower	49		1.00				1.00		
Tertiles	Middle	49		3.77	1.49	9.51		2.73	.89	8.40
	Upper	49		5.81	2.38	14.14		3.46	1.10	10.86
		***TREND:***	***χ2 =***	***16.54***	***P-value =***	***.0000***	***χ2 =***	***4.00***	***P-value =***	***.0454***
**TLG-0**	Lower	49		1.00						
Tertiles	Middle	49		2.96	1.24	7.10				
	Upper	49		4.90	2.11	11.38				
		***TREND:***	***χ2 =***	***14.95***	***P-value =***	***.0001***				
**SUVmax-2**	Lower	49		1.00				1.00		
Tertiles	Middle	49		1.40	.65	2.99		2.56	.20	33.22
	Upper	49		2.46	1.22	4.96		0.98	.06	16.10
		***TREND:***	***χ2 =***	***6.72***	***P-value =***	***.0096***	***χ2 =***	***2.40***	***P-value =***	***.1216***
**MTV-2**	Lower	59		1.00				1.00		
Tertiles	Middle	39		1.49	.68	3.27		4.16	.49	35.29
	Upper	49		3.09	1.58	6.05		8.08	.72	90.67
		***TREND:***	***χ2 =***	***11.21***	***P-value =***	***.0008***	***χ2 =***	***3.02***	***P-value =***	***.0820***
**TLG-2**	Lower	59		1.00						
Tertiles	Middle	39		1.49	.68	3.27				
	Upper	49		3.09	1.58	6.05				
		***TREND:***	***χ2 =***	***11.21***	***P-value =***	***.0008***				
**ΔSUVmax**	Lower	49		1.00				1.00		
Tertiles	Middle	49		0.97	.46	2.07		1.04	.32	3.31
	Upper	49		2.16	1.11	4.21		1.16	.32	4.17
		***TREND:***	***χ2 =***	***5.42***	***P-value =***	***.0199***	***χ2 =***	***0.09***	***P-value =***	***.7701***
**ΔMTV**	Lower	59		1.00						
Tertiles	Middle	39		2.63	1.30	5.33				
	Upper	49		2.02	.99	4.12				
		***TREND:***	***χ2 =***	***3.83***	***P-value =***	***.0502***				
**ΔTLG**	Lower	59		1.00						
Tertiles	Middle	39		2.66	1.31	5.40				
	Upper	49		2.00	.98	4.07				
		***TREND:***	***χ2 =***	***3.71***	***P-value =***	***.0542***				

MTV and TLG measures correlated very strongly at both baseline and after two cycles (Pearson’s correlation coefficient, *r* = 0.916 and *r* = 0.961, respectively), as tertile groups were almost identical. Therefore, only MTV was included in multivariate analysis (MVA). The MVA included IPI, DS, MTV-0, MTV-2, SUVmax-2 and ΔSUVmax, and showed that MTV-0 was the only independent statistically significant measure.

**Fig. 2:**
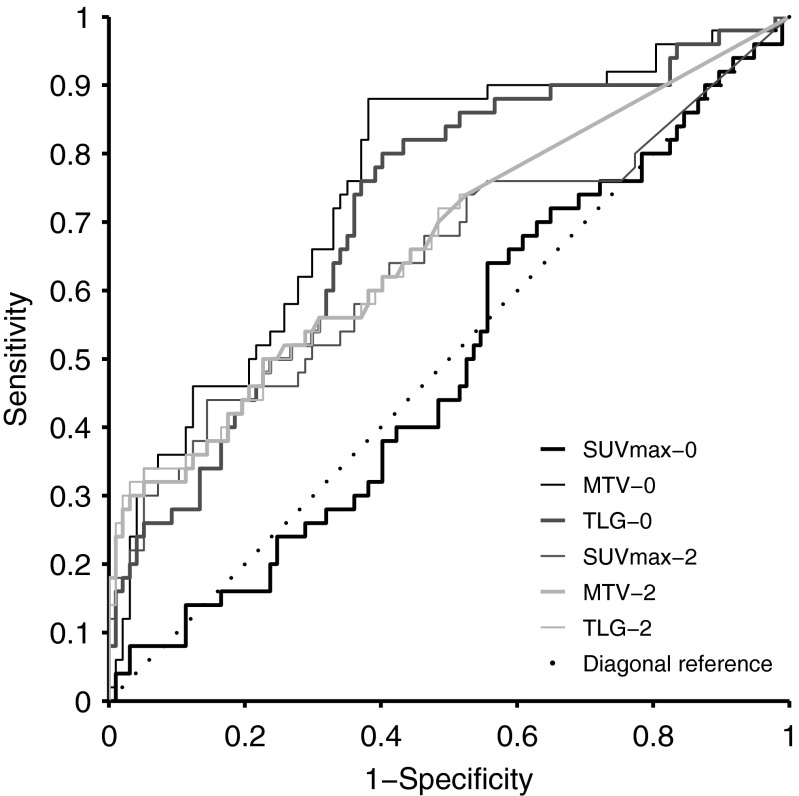
Receiver operator characteristics analysis

ROC analysis (Fig. 2) showed that the optimal cut-off for sensitivity and specificity for MTV-0 was 396 cm^3^ (sensitivity 0.88, specificity 0.62) and for TLG-0 was 4541 (sensitivity 0.76, specificity 0.63). The optimal cut-offs for MTV-2 and TLG-2 were skewed towards very low values (1.8 and 5.6 respectively), reflecting the fact that most patients had significant reduction in FDG activity after two cycles of chemotherapy. Similarly, the cut-offs for ΔMTV and ΔTLG were 99.76 % and 99.9 %. Therefore, only the cut-offs for MTV-0 and TLG-0 were considered clinically meaningful.

**Fig. 3:**
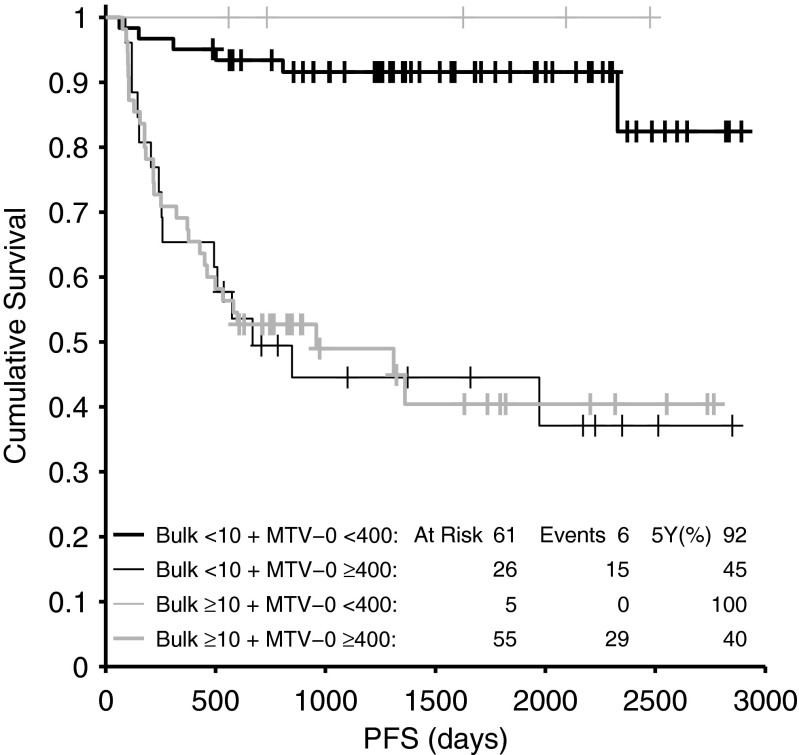
Relationship between initial bulk and MTV-0

We also examined the relationship between the presence of bulky disease (defined as maximum tumour dimension (MTD) in any direction ≥10 cm as defined by ROC analysis, data not shown) and MTV-0. Overall, 59 patients (40 %) had bulky disease. As expected, the presence of bulky disease correlated with high MTV-0 (parametric Pearson's correlation coefficient, *r* = 0.557, *p* < 0.001). However, MTV-0 predicted the outcome of patients better than MTD. Overall concordance was 79 % (41 % had low bulk and low MTV-0, and 38 % had high bulk and high MTV-0). Thirty-one patients (21 %) had discordance of bulk and MTV-0 classification. Twenty-six patients (18 %) had low bulk but high MTV-0, and their 5-year PFS was 45 %. Five patients (3 %) had high bulk but low MTV-0, and their 5-year PFS was 100 % (Fig. 3).

**Fig. 4:**
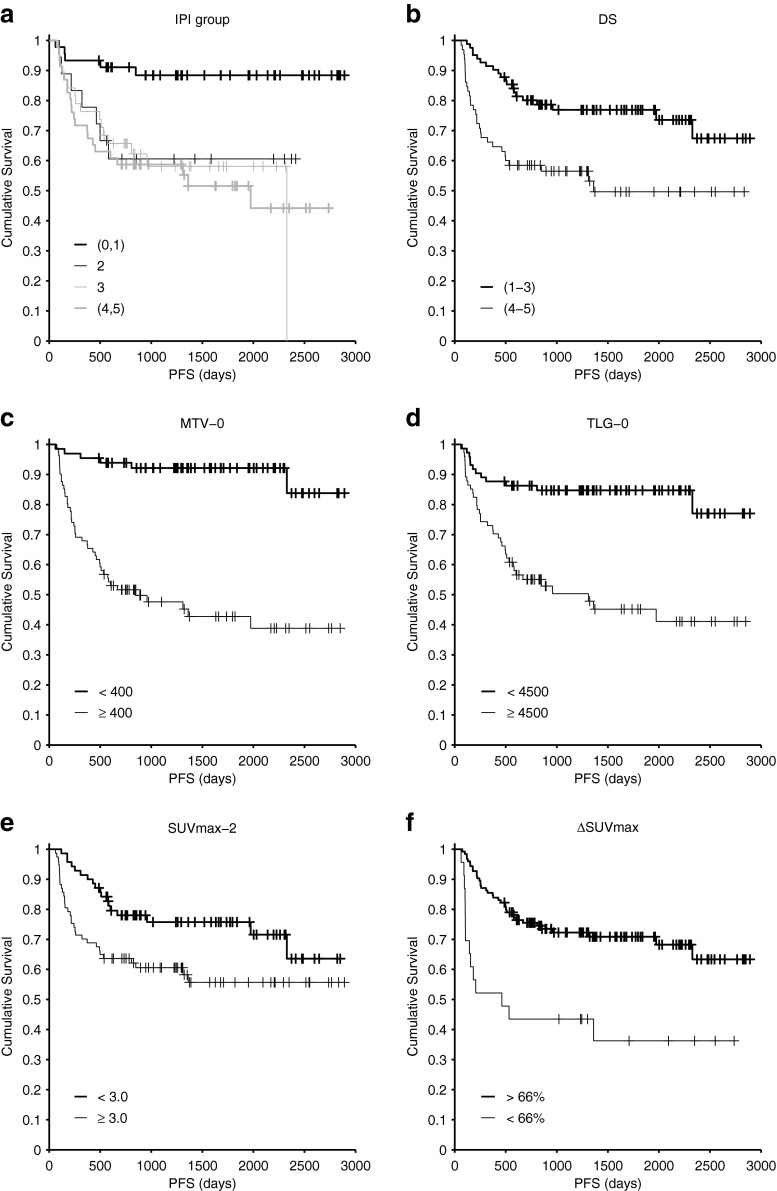
Kaplan–Meier progression-free survival curves. **a**: IPI groups **b**: DS **c:** MTV-0 **d**: TLG-0 **e**: SUVmax-2 **f**: ΔSUVmax

KM survival analysis showed that the following variables were associated with PFS: IPI (0–1 v 2–5: X^2^ = 14.17, *p* < 0.001), MTV-0 (<400 vs. >400: X^2^ = 34.17, *p* < 0.001), TLG-0 (<4500 vs. >4500: X^2^ = 20.13, *p* < 0.001), DS (1–3 vs. 4–5: X^2^ = 10.51, *p* = 0.001), MTV-2 (<1.8 vs. >1.8: X^2^ = 11.32, *p* = 0.001), TLG-2 (<5.6 vs. >5.6: X^2^ = 10.36, *p* = 0.001), SUVmax-2 (<3 vs. >3: X^2^ = 5.3, *p* = 0.021), ΔMTV (>99.8 % vs. <99.8 %: X^2^ = 6.36, *p* = 0.012), ΔTLG (>99.9 % vs. <99.9 %: X^2^ = 7.43, *p* = 0.006), ΔSUVmax (>66 % vs. <66 %: X^2^ = 13.68, *p* < 0.001). Figure 4 shows examples of KM PFS analysis.

For overall survival analysis, the corresponding values were: IPI (0–1 vs. 2–5: X^2^ = 18.4, *p* < 0.001), MTV-0 (<400 vs. >400: X^2^ = 23.9, *p* < 0.001), TLG-0 (<4500 vs. >4500: X^2^ = 12.8, *p* < 0.001), DS (1–3 vs. 4–5: X^2^ = 7.13, *p* = 0.008), MTV-2 (<1.8 vs. >1.8: X^2^ = 4.1, *p* = 0.043), TLG-2 (<5.6 vs. >5.6: X^2^ = 3.65, *p* = 0.056), SUVmax-2 (<3 vs. >3: X^2^ = 2.79, *p* = 0.095), ΔMTV (>99.8 % vs. <99.8 %: X^2^ = 3.06, *p* = 0.08), ΔTLG (>99.9 % vs. <99.9 %: X^2^ = 2.81, *p* = 0.094), ΔSUVmax (>66 % vs. <66 %: X^2^ = 6.49, *p* = 0.011).

**Fig. 5:**
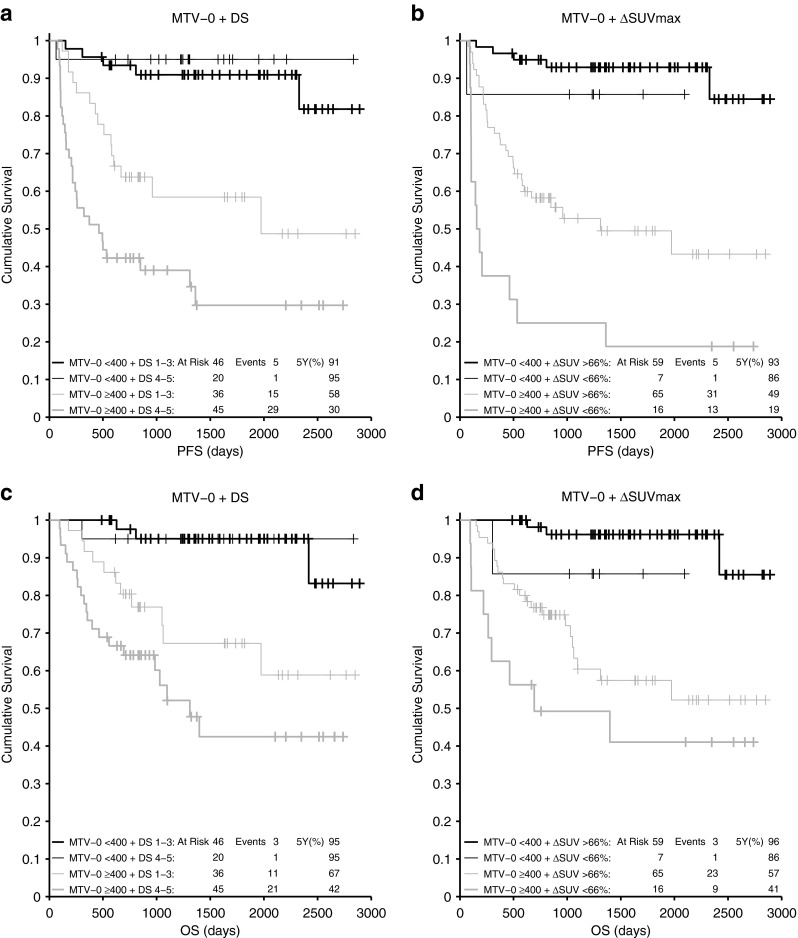
Prognostic model **a**: Model combining MTV-0 + DS (PFS). **b**: Model combining MTV-0 + ΔSUVmax (PFS). **c**: Model combining MTV-0 + DS (OS). **d**: Model combining MTV-0 + ΔSUVmax (OS)

To test the study hypothesis that a combination of baseline metabolic tumour burden and response after two cycles can improve the prognostic ability of iPET-2 in DLBCL, we constructed a prognostic model combining MTV-0—the only statistically significant baseline measure on MVA—and DS. The aim was to identify a group with sufficiently poor prognosis that clinicians might consider changing treatment, and large enough to include most of the population events. Patients were classified into four groups according to the two parameters: MTV-0 < 400 + DS 1–3 (46 patients, 31 %), MTV-0 < 400 + DS 4–5 (20 patients, 14 %), MTV-0 ≥ 400 + DS 1–3 (36 patients, 24 %) and MTV-0 ≥ 400 + DS 4–5 (45 patients, 31 %) (Fig. 5a).

This model showed that patients with high baseline tumour burden (MTV-0 ≥ 400) and poor response (DS 4–5) had a much worse prognosis than the remainder of the study population with 5-year PFS 29.7 %. Patients with low MTV-0 had a favourable prognosis regardless of the DS after two cycles (5-year PFS 90.9 and 95.0 %), and patients with high MTV-0 who responded well according to DS had an intermediate prognosis (5-year PFS 58.5 %). The worst prognosis group included 31 % of the study population but 58 % of the total events (29/50). Hazard ratios (compared to the group with low MTV-0 and low DS) were 0.49 (low MTV-0 + high DS, 95 % CI 0.06-4.21), 4.7 (high MTV-0 + low DS, 95 % CI 1.7-13.0) and 10.1 (high MTV-0 + high DS, 95 % CI 3.9-26.2).

We examined other models—e.g. replacing MTV-0 with TLG-0 or IPI, replacing DS with ΔSUVmax or adding other parameters to MTV-0 and DS—but none was able to predict a worse prognostic group, and the current model remained the simplest and most predictive. The model combining MTV-0 and ΔSUVmax, for example, is shown in Fig. 5b. The worst prognosis group included only 11 % of patients and 26 % of the events.

## Discussion

The addition of rituximab to CHOP chemotherapy has improved the outcome of first-line treatment for DLBCL [1, 2], but salvage therapy for patients who are not cured with RCHOP is less effective than that after CHOP [3, 4]. Early identification of patients who are unlikely to be cured with RCHOP is an important step to enable testing of alternative approaches. Although early response assessment using iPET has been established in Hodgkin lymphoma, its role remains controversial in DLBCL due to conflicting results [10–21] and the inability to detect a group with sufficiently poor prognosis even with new response criteria such as DS or ΔSUVmax [25]. For example, a recently published study using DS showed a statistically significant difference between scores 1–3 and 4–5 (85 % vs. 72 % respectively, p = 0.047), but the worse group still had excellent PFS [18]. Another study using ΔSUVmax also showed a statistically significant difference between the two groups, while the worse prognostic group still had a high PFS [17].

In order to improve the predictive ability of PET, we assessed the prognostic value of various quantitative PET parameters, and found that the pre-treatment total metabolic burden was the strongest predictor of PFS. MTV-0 and TLG-0 were the most predictive quantitative measures on univariate analysis and were superior to the standard tools for assessing pre-treatment risk and early response, namely the IPI and DS. As results using MTV and TLG were almost identical, we included only MTV parameters in MVA, which showed that MTV-0 remained statistically significant while IPI and DS did not. These results confirm previous studies showing the prognostic value of baseline metabolic tumour burden [26–29]; however, this is the first study to show that pre-treatment total metabolic burden is more prognostic than either early response or IPI in DLBCL.

We also examined the relationship between the presence of bulky disease (as defined by anatomical size criteria) and MTV-0. Bulk has traditionally been considered a poor prognostic factor, although the IPI did not include bulky disease [22]. The prognostic value of bulk was demonstrated in the rituximab era, and the optimal cut-off appeared to be 10 cm (measuring the maximum tumour dimension = MTD) in RCHOP-treated patients [30]. Measuring MTD is technically easier and quicker than measuring MTV, so we compared the two parameters to determine which was more predictive and whether bulk expressed as MTD could predict the same outcome while being easier to measure. Using MTD cut-off of ≥10 cm, 59 patients (40 %) were found to have bulky disease. As expected, the presence of bulky disease correlated with high MTV-0; however, MTV-0 predicted the outcome of patients much better than MTD. Overall, the two parameters agreed in 79 % of patients, but in 21 % of patients who had discordance of bulk and MTV-0 classification, MTV-0 correctly predicted the outcome regardless of the presence or absence of bulk. Twenty-six patients (18 %) had low bulk but high MTV-0, and their 5-year PFS was 45 %, while five patients (3 %) had high bulk but low MTV-0, and their 5-year PFS was 100 %.

While baseline metabolic burden was a very strong predictor in this population, the change in metabolic burden was less predictive. The percentage reductions in MTV and TLG after two cycles (ΔMTV and ΔTLG, respectively) were borderline statistically significant on univariate analysis. On ROC analysis, the optimal cut-offs for ΔMTV and ΔTLG were skewed (99.76 % and 99.9 %, respectively), indicating that most of the population had a significant reduction in metabolic volume after two cycles of chemotherapy. Similarly, the optimal cut-offs for MTV-2 and TLG-2 were skewed towards very low values (1.8 and 5.6, respectively).

On the other hand, ROC analysis showed that the optimal cut-off for sensitivity and specificity for MTV-0 was 396 cm^3^ (rounded to 400) and for TLG-2 was 4541 cm^3^ (rounded to 4500). These were considered clinically meaningful and were included in further analysis. Using these cut-offs, KM survival analysis separated the study population into two distinct prognostic groups with statistically significant differences in PFS.

Other studies have reported optimal cut-offs for MTV-0 ranging from 220 to 625 cm^3^ [26, 27, 29]. The optimal cut-off value is dependent on the characteristics of the study population and the methodology of MTV measurement. Studies with populations with better prognosis (e.g. earlier stage, less bulky disease and/or low IPI) resulted in lower cut-offs and vice versa. Song et al. [26] studied only stage 2–3 patients without extra-nodal involvement and few (4.1 %) with bulky disease (>5 cm), and reported the optimal cut-off as 220 cm^3^. Sasanelli et al. [29] included patients from eight institutions, most with stage 3–4 (82 %), with bulky disease (>10 cm) in 36 % and two or more extra-nodal sites in 32 %, and found an optimal cut-off of 550 cm^3^. Our study was non-selective and included consecutive patients from one institution with all stages and presentations of disease, and so is likely more representative of the general population of DLBCL in clinical practice. Interestingly, the optimal cut-off in our study was in the middle of the wide reported range. The other factor affecting MTV-0 cut-off is the measurement method. We applied the method used by Song et al. [26], defining the MTV by SUVmax cut-off of 2.5, while Sasanelli [29] and Casanovas [27] used a threshold of 41 % of SUVmax in each lesion to define MTV. Meignan et al. [31] recently published a study evaluating different methods using phantom and patient data, and concluded that 41 % is the optimal cut-off to define MTV in lymphomas, in line with solid tumours [32]. Our study, however, was conducted before this recent publication. Validation studies comparing the prognostic ability of the two methods are needed to resolve this issue, but it seems that in the current study population, the MTV-0 cut-off of 400 cm^3^, defined by SUVmax > 2.5, resulted in a clinically significant separation of low- and high-MTV-0 groups (3-year PFS 92.2 % vs. 47.6 %) and compares favourably to MTV-0 defined by 41 % of SUVmax (3-year PFS 77 % vs. 60 %) [29].

One of the study objectives was to test the hypothesis that a combination of baseline measurement of metabolic tumour burden and early response assessment could improve the prognostic ability of iPET-2 in DLBCL. The model combining MTV-0 and DS was able to separate significantly different prognostic groups and to identify a group with a prognosis sufficiently poor that clinicians might consider changing treatment. Patients with high baseline tumour burden (MTV-0 ≥ 400) and poor response after two cycles (DS 4–5) had a much worse prognosis than the remainder of the study population with 5-year PFS of 29.7 %. This compares favourably with either DS alone (5-year PFS of poor prognosis group 49.6 %) or ΔSUVmax alone (5-year PFS of poor prognosis group 36.2 %, but this group included only 16 % of patients). Patients with low MTV-0 had a favourable prognosis regardless of their DS after two cycles (5-year PFS 90.9 and 95.0 %), indicating that pre-treatment prognosis is more important than early response in this group. Conversely, patients with high MTV-0 who responded well according to DS had an intermediate prognosis (5-year PFS 58.5 %). It is also worth noting that MTV-0 separated the DS 4–5 patients into the best and the worst prognostic groups, and correctly reclassified 20 patients as good prognosis with 5-year PFS 95 %. Finally, the worst prognosis group included 58 % of the total number of events (29/50) and 31 % of patients, making this model useful for identifying patients with poor prognosis.

Our results have significant clinical implications, and the proposed prognostic model based on both initial metabolic burden and response after two cycles of chemotherapy could be used in both clinical practice and future clinical trials. Most notably, the separation of poor early responders (DS 4–5) into very different prognostic groups (best and worst) depending on MTV-0 can potentially be used to test response-adapted treatments. However, the proposed model must be validated in another independent data set to confirm its prognostic value and optimal cut-off, and to test it against MTV defined by 41 % cut-off.

The recently published imaging guidelines (ICML guidelines) [23] and Lugano classification [33] now recommend performing PET/CT for staging in all FDG-avid lymphomas, and baseline scan information should be readily available. In addition, efforts to standardise PET methods to ensure quantitative accuracy in everyday practice [32, 34] and the increasing availability of automated software from manufacturers to measure metabolic volumes make quantitative analysis in the clinic a realistic option in the near future.

## Conclusions

Baseline metabolic tumour volume is an important prognostic factor in diffuse large B-cell lymphoma treated with RCHOP chemotherapy. The combination of baseline metabolic tumour volume and response on PET/CT after two cycles measured by Deauville score improves the predictive power of interim PET and separates patients into distinct groups with different prognosis, defining a poor-prognosis group which includes most of the events after treatment.

*IPI*, international prognostic index; *DS*, Deauville score after 2 cycles; *SUVmax*-*0*, baseline maximum standardised uptake value; *MTV*-*0*, baseline metabolic tumour volume; *TLG*-*0*, baseline total lesion glycolysis; *SUVmax-2*, maximum standardised uptake value after 2 cycles; *MTV-2*, metabolic tumour volume after 2 cycles; *TLG-2*, total lesion glycolysis after 2 cycles; *ΔSUVmax*, percentage change in maximum standardised uptake value; *ΔMTV*, percentage change in metabolic tumour volume; *ΔTLG*, percentage change in total lesion glycolysis; *ROC*, receiver operator characteristics
